# Genome‐wide association and polygenic risk score estimation of type 2 diabetes mellitus in Kinh Vietnamese—A pilot study

**DOI:** 10.1111/jcmm.18526

**Published:** 2024-07-03

**Authors:** Thao Phuong Mai, Bac An Luong, Phat Tung Ma, Thang Viet Tran, Tat Thang Dinh Ngo, Chi Khanh Hoang, Luong Van Tran, Bao Hoang Le, Hoang Anh Vu, Linh Hoang Gia Le, Khuong Thai Le, Steven Truong, Nam Quang Tran, Minh Duc Do

**Affiliations:** ^1^ Department of Physiology‐Pathophysiology‐Immunology, Faculty of Medicine University of Medicine and Pharmacy at Ho Chi Minh City Ho Chi Minh City Vietnam; ^2^ Center for Molecular Biomedicine University of Medicine and Pharmacy at Ho Chi Minh City Ho Chi Minh City Vietnam; ^3^ Department of Endocrinology, Faculty of Medicine University of Medicine and Pharmacy at Ho Chi Minh City Ho Chi Minh City Vietnam; ^4^ Department of Endocrinology University Medical Center Ho Chi Minh City Ho Chi Minh City Vietnam; ^5^ MIT Department of Biological Engineering Cambridge Massachusetts USA

**Keywords:** genetic, GWAS, Kinh Vietnamese, type 2 diabetes mellitus

## Abstract

A genome‐wide association study (GWAS) is a powerful tool in investigating genetic contribution, which is a crucial factor in the development of complex multifactorial diseases, such as type 2 diabetes mellitus. Type 2 diabetes mellitus is a major healthcare burden in the Western Pacific region; however, there is limited availability of genetic‐associated data for type 2 diabetes in Southeast Asia, especially among the Kinh Vietnamese population. This lack of information exacerbates global healthcare disparities. In this study, 997 Kinh Vietnamese individuals (503 with type 2 diabetes and 494 controls) were prospectively recruited and their clinical and paraclinical information was recorded. DNA samples were collected and whole genome genotyping was performed. Standard quality control and genetic imputation using the 1000 Genomes database were executed. A polygenic risk score for type 2 diabetes was generated in different models using East Asian, European, and mix ancestry GWAS summary statistics as training datasets. After quality control and genetic imputation, 107 polymorphisms reached suggestive statistical significance for GWAS (≤5 × 10^−6^) and rs11079784 was one of the potential markers strongly associated with type 2 diabetes in the studied population. The best polygenic risk score model predicting type 2 diabetes mellitus had AUC = 0.70 (95% confidence interval = 0.62–0.77) based on a mix of ancestral GWAS summary statistics. These data show promising results for genetic association with a polygenic risk score estimation in the Kinh Vietnamese population; the results also highlight the essential role of population diversity in a GWAS of type 2 diabetes mellitus.

## 1 INTRODUCTION

Type 2 diabetes mellitus (T2DM) is a significant healthcare burden worldwide.^1^ According to the estimation of International Diabetes Federation, the prevalence of diabetes in Vietnam ranges from 6.1% to 7.1% and 90% of the diabetic cases are type 2.^2^ Like other complex diseases, such as cardiovascular diseases and neurogenerative diseases, T2DM is multifactorial, with both genetic and environmental factors playing crucial roles in the development of the disease.^3^, ^4^, ^5^ It has been shown that family history significantly increases the risk of developing T2DM, suggesting heritability of the disease.^6^, ^7^

Understanding the genetic contribution to T2DM is essential, as it provides useful information for T2DM prediction and prevention. A powerful tool that can be used to obtain genetic insights into complex diseases such as T2DM is a genome‐wide association study (GWAS). To date, most GWAS results for T2DM relate to Caucasian and East Asian populations, although the large populations in Southeast Asia also have significant levels of T2DM and have been found to be genetically susceptible to T2DM.^8^, ^9^ The lack of T2DM‐related GWAS data not only hinders the application of these results in Asia but also exacerbates healthcare disparities. Therefore, the need for GWAS data on T2DM in Southeast Asian populations is strongly required.

Located in Southeast Asia, Vietnam is one of the most populated countries in the Western Pacific region, with more than 100 million inhabitants. Kinh Vietnamese account for more than 80% of the total population. Before 2022, only a small, single GWAS study had been performed on Kinh Vietnamese to investigate the genetic basis for schizophrenia; there is no available GWAS data for T2DM or cardiovascular diseases.^10^ GWAS results are much more reliable with larger sample sizes, and the first GWAS conducted on a Vietnamese population used more than 300 subjects.^10^ Our aim was to perform the first GWAS on T2DM in Kinh Vietnamese. Briefly, we recruited 503 T2DM cases and 494 controls, the participants' DNA samples were used to perform genome‐wide genotyping. The output data underwent quality control, genotype imputation, and genetic association analysis. Polygenic risk score (PRS) for T2DM was also calculated. These early findings should provide further information on potential novel genetic associations and PRS estimations for T2DM, but the wider aim is to provide genetic data on Kinh Vietnamese that can be used in future GWAS meta‐analyses.

## 2 MATERIALS AND METHODS

### 2.1 Subject recruitment

The study protocol was approved by the Ethical Committee of the University of Medicine and Pharmacy in Ho Chi Minh City (Approval number: 350/HDDD‐DHYD). Recruitment took place from May 2020 to September 2021 in the University Medical Center of the University of Medicine and Pharmacy. The participants all self‐identified as Kinh Vietnamese and signed the informed written consent. Two sample sets were collected for this study: a T2DM set and a control set. Subjects with T2DM were recruited if they had either a history of T2DM or were newly diagnosed with T2DM, based on the American Diabetes Association 2020 criteria.^11^ T2DM cases were excluded from the study if they had type 1 diabetes or elevated plasma glucose due to endocrine diseases or drug usage. The controls were recruited randomly among visitors attending for a health check‐up who had no history of diabetes and who had normal plasma glucose and HbA1c levels. The exclusion criteria for the controls were a history of diabetes, pregnancy, or cancer.

All participants provided basic clinical information and underwent a detailed physical examination. Demographic information (age, sex), duration of T2DM, and anthropometric parameters (weight, height, waist circumference, hip circumference, and systolic and diastolic blood pressure) were documented. Laboratory data were obtained from fasting (minimum 8 h) blood samples for all participants using a Beckman Coulter AU2700 Chemistry Analyzer (Beckman Coulter, California, USA). Fasting plasma glucose, HbA1c, total serum cholesterol, high‐density lipoprotein (HDL) cholesterol, low‐density lipoprotein (LDL) cholesterol, triglycerides, and creatinine levels were recorded. Each participant also provided 2 mL of peripheral venous blood for genetic analysis.

### 2.2 Genotyping and analysis

#### 2.2.1 Genome‐wide genotyping

Genomic DNA was extracted from 997 peripheral whole blood samples using a QIAGEN Blood Mini Kit (QIAGEN, Hilden, Germany). DNA samples were quantified using a Qubit™ 3 Fluorometer (Thermo Fisher Scientific, Waltham, MA, USA) and Qubit™ dsDNA Quantification Assay Kits (Thermo Fisher Scientific). Genome‐wide genotyping of the DNA samples was performed using an Infinium Global Screening Array‐24 v2.0 BeadChip (Illumina, California, USA) following the manufacturer's protocol. The intensity files (.idat), which are the raw data files from the BeadChip, were read by GenomeStudio (v2.0) (Illumina, San Diego, CA). The primary filter criteria were set up with Call Rate ≥0.98, GenTrain Score >0.7, Cluster Sep Score >0.3, and Call Freq ≥0.95. Qualified samples were exported in PLINK format as .ped and .map files.

#### 2.2.2 Quality control

Quality control on the samples was carried out using PLINK1.9 with default parameters. The criteria for single nucleotide polymorphisms (SNPs) and individual quality data included individual missingness <0.03; sex discrepancy (X chromosome heterozygous/homozygous rate is <0.2 for females and >0.8 for males); minor allele frequency (MAF) >0.05; SNP missingness <0.01; Hardy–Weinberg equilibrium (HWE) *p* > 10^−6^ (both case and control); heterozygosity rate ± 3 SD from the samples' mean heterozygosity rate; and related individuals identified by descent pi‐hat <0.1875 and ethnic outliers (non‐East Asian ancestry).

#### 2.2.3 Genotype imputation

Before conducting the imputation, the dataset was pre‐phased using SHAPEIT2 (University of Oxford).^12^ Genotype imputation was performed using the imputation stepwise approach implemented in IMPUTE2 (with default parameters),^13^ specific parameters included buffer size 250 kb, imputing into an East Asian dataset, and allele frequency >0.01 to reduce the cost of computing. The imputation reference data were 1000 Genomes haplotype phase 3 with 77,818,332 SNPs.

#### 2.2.4 Genetic association analysis

Analysis of single SNP associations was carried out using SNPTEST v.2.5.6 (University of Oxford).^14^ After imputation, SNPs with imputation quality (INFO) <0.8 were excluded prior to genetic analyses. The genome‐wide association was illustrated by Manhattan and QQ plots. For association analysis, *p*‐value ≤5 × 10^−8^ was considered statistical significance, while *p*‐value ≤5 × 10^−6^ was considered suggestive statistical significance. Besides Bonferroni correction, Benjamini–Hocberg procedure was also applied to adjust *p*‐value for multiple comparisons with a false discovery rate of 20%.

#### 2.2.5 Polygenic risk scoring

PRS analysis was performed using the PRSice‐2 package.^15^ Briefly, the approach constructs a polygenic score by summing all trait‐associated alleles in a target sample, weighted by the effect size of each allele in a base GWAS. We obtained summary statistics (base GWAS) with effect sizes of associated SNPs from the GWAS Catalogue of the European Bioinformatics Institute to calculate PRS for individuals in our target dataset.^16^ Genome‐wide summary statistics were ‘clumped’ by various thresholds of *p*‐value significance (5 × 10^−2^; 5 × 10^−4^; 5 × 10^−6^; 5 × 10^−8^) within 250 kb and –clump‐*r* ^2^ = 0.2. Different ethnic datasets (base) summary statistics for the T2DM trait in the GWAS Catalogue were used: (i) East‐Asian (EAS) specific meta‐analysis (77,418 cases and 356,122 controls),^17^ (ii) European (EUR) specific meta‐analysis (26,676 cases and 132,532 controls),^18^ and (iii) mix ancestral (MIX) meta‐analysis (84,244 cases and 583,280 controls).^19^ We built models based on logistic regression to make predictions. In simulation studies, logistic regression was performed using PRS as an independent variable. Our samples were split into training and testing datasets, and used three ratios, 80:20, 50:50, and 20:80. The performance of the prediction models was assessed by the area under the curve (AUC) and confidence intervals of AUC using the pROC package within R.

#### 2.2.6 Statistical analysis

Frequencies and percentages were used to express qualitative variables. Quantitative variables were tested for normal distribution using the Kolmogorov–Smirnov test, and are presented as mean with standard deviation (SD) if normally distributed or median with interquartile range [Q1–Q3] if not normally distributed. Differences in characteristics were compared using chi‐squared for qualitative variables and Student's *t*‐test or the Mann–Whitney *U*‐test for quantitative variables, based on the data distribution. SPSS Statistics for Windows version 20.0 (IBM Corp., Armonk, NY, USA) was used for statistical analysis. A *p*‐value of <0.05 was considered to be statistically significant.

## 3 RESULTS

### 3.1 Characteristics of the recruited samples

A total of 997 qualified samples were used, with 503 cases of T2DM and 494 controls. The demographic characteristics of all the participants are described in Table 1. Notably, the age of T2DM onset in the T2DM group was significantly lower than the age at recruitment of the control group. The T2DM group also had significantly greater BMI and waist–hip ratio compared to the control group.

**TABLE 1:** Baseline characteristics of the participants.

Characteristics	Total *N* = 997	T2DM *N* = 503	Control *N* = 494	*p*‐value
Age at recruitment (year) (mean ± SD)	50.2 ± 12.6	51.2 ± 12.6	49.1 ± 12.6	<0.01
Age of T2DM onset (T2DM)/age at recruitment (control) (year) (mean ± SD)	47.6 ± 12.1	46.0 ± 11.9	49.1 ± 12.6	<0.001
Duration of T2DM (year) (median [Q1–Q3])	–	3 [1–8]	–	–
Gender (*n*, %)				
Male	402 (40.3)	223 (44.3)	179 (36.2)	0.01
Female	595 (59.7)	280 (55.7)	315 (63.8)	
Family history of T2DM (*n*, %)				
Yes	323 (32.4)	219 (43.5)	104 (21.1)	<0.001
No	674 (67.6)	284 (56.5)	390 (78.9)	
Height (cm) (mean ± SD)	159.1 ± 7.6	159.1 ± 7.6	159.0 ± 7.7	0.72
Weight (kg) (mean ± SD)	61.2 ± 10.9	63.3 ± 11.4	59.2 ± 10.1	<0.001
BMI (kg/m^2^) (mean ± SD)	24.1 ± 3.7	24.9 ± 3.8	23.4 ± 3.4	<0.001
Waist circumference (cm) (mean ± SD)	84.0 ± 10.2	86.6 ± 10.3	81.4 ± 9.4	<0.001
Hip circumference (cm) (mean ± SD)	92.7 ± 8.5	94.3 ± 8.4	91.1 ± 8.4	<0.001
Waist–hip ratio (mean ± SD)	0.91 ± 0.07	0.92 ± 0.07	0.89 ± 0.07	<0.001
SBP (mmHg) (mean ± SD)	129.4 ± 17.1	130.8 ± 17.7	127.9 ± 16.4	0.01
DBP (mmHg) (mean ± SD)	10.0 ± 10.8	79.7 ± 11.0	80.3 ± 10.6	0.40
FPG (mmol/L) (mean ± SD)	7.3 ± 3.1	9.0 ± 3.5	5.6 ± 0.8	<0.001
HbA1c (%) (mean ± SD)	7.8 ± 2.2	8.3 ± 2.1	5.7 ± 0.5	<0.001
eGFR (mL/min/1.73 m^2^) (mean ± SD)	85.3 ± 20.2	84.2 ± 20.7	86.5 ± 19.5	<0.001
Total cholesterol (mmol/L) (mean ± SD)	5.1 ± 1.5	4.8 ± 1.5	5.4 ± 1.4	<0.001
LDL‐cholesterol (mmol/L) (mean ± SD)	3.2 ± 1.0	2.9 ± 1.0	3.5 ± 1.0	<0.001
HDL‐cholesterol (mmol/L) (mean ± SD)	1.2 ± 0.4	1.1 ± 0.4	1.3 ± 0.5	<0.001
Triglyceride (mmol/L) (median [Q1–Q3])	1.8 [1.2–2.8]	1.9 [1.4–3.1]	1.6 [1.1–2.4]	<0.001

Abbreviations: BMI, body mass index; DBP, diastolic blood pressure; eGFR, estimated glomerular filtration rate; FPG, fasting plasma glucose; HDL, high‐density lipoprotein; LDL, low‐density lipoprotein; SBP, systolic blood pressure; SD, standard deviation; T2DM, type 2 diabetes mellitus.

### 3.2 Quality control

After inputting raw data for the 997 samples to GenomeStudio, six samples with Call Rate <0.98 were excluded; 991 samples with 665,539 SNPs each were exported in PLINK format containing .ped and .map files. Twenty‐four samples which did not meet quality control (QC) criteria were further excluded from the analysis. Finally, 957 samples with 379,428 SNPs each passed the QC, and these results were used for genotype imputation. After imputation, 957 samples with 7,587,388 SNPs each were used for genetic association analysis.

### 3.3 Genetic association analysis

**FIGURE 1:**
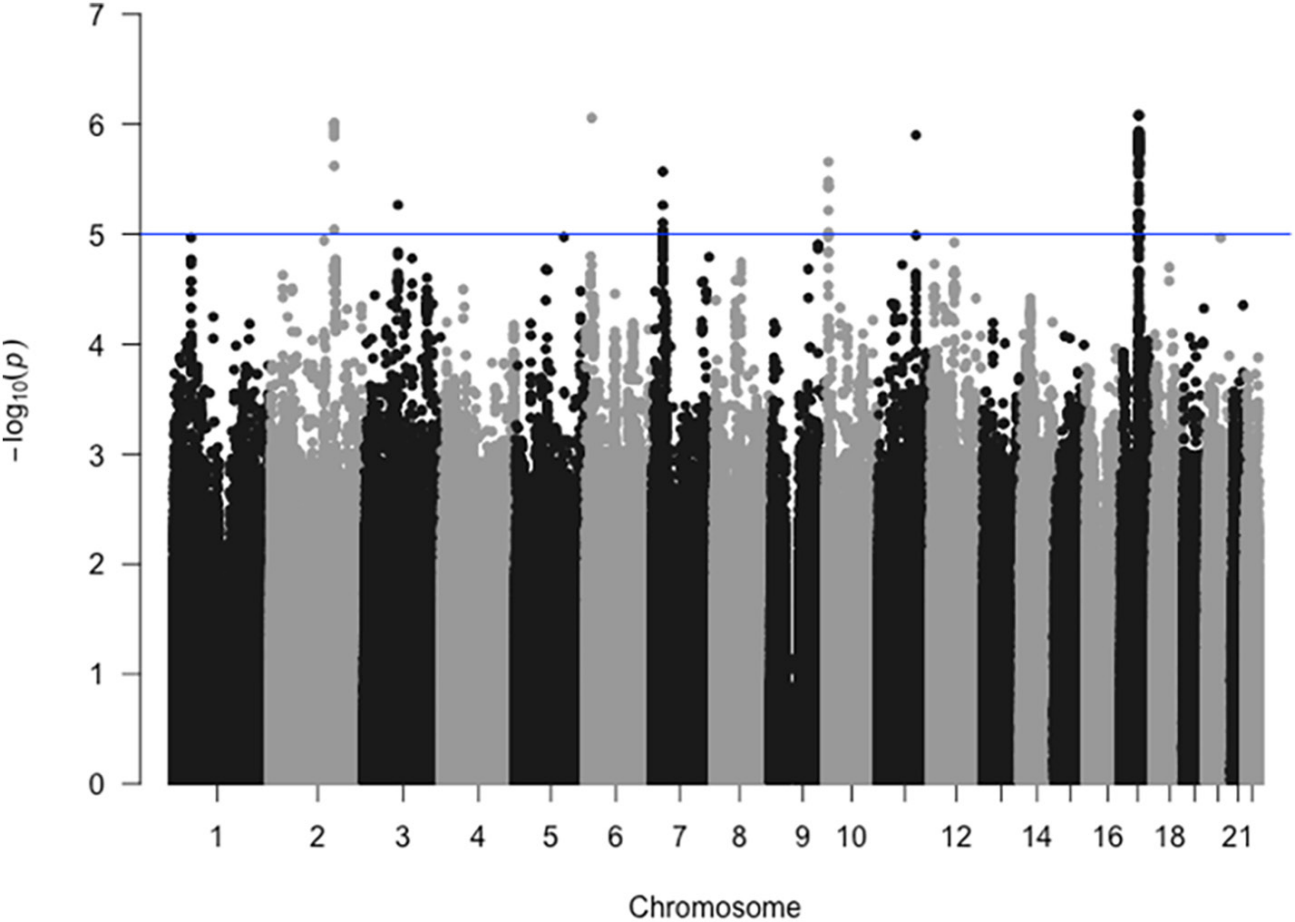
Manhattan plot after quality control. Chromosome position in ascending order is indicated on the *x* axis, the −log10 (*p*‐values) of included autosomal SNPs derived from the association analysis are plotted on the *y* axis.

Before imputation, SNP tests showed that rs11079784 was the strongest SNP associated with T2DM (*p*‐value = 8.8 × 10^−7^) with OR = 1.58. After imputation, the results of the SNP tests showed that 107 SNPs reached the suggestive threshold of statistical significance for GWAS (*p*‐value ≤5 × 10^−6^). These SNPs are mainly located in chromosomes 2, 6, 10, 11, and 17. By using Benjamini–Hochberg correction, there were 37 SNPs statistically associated with T2DM and these SNPs are mainly located in chromosome 17. Post‐QC Manhattan and QQ plots did not show any unexpected genome‐wide significant associations (Figure 1). A comprehensive list of these associated SNPs is in Table S1.

### 3.4 PRS calculation

**FIGURE 2:**
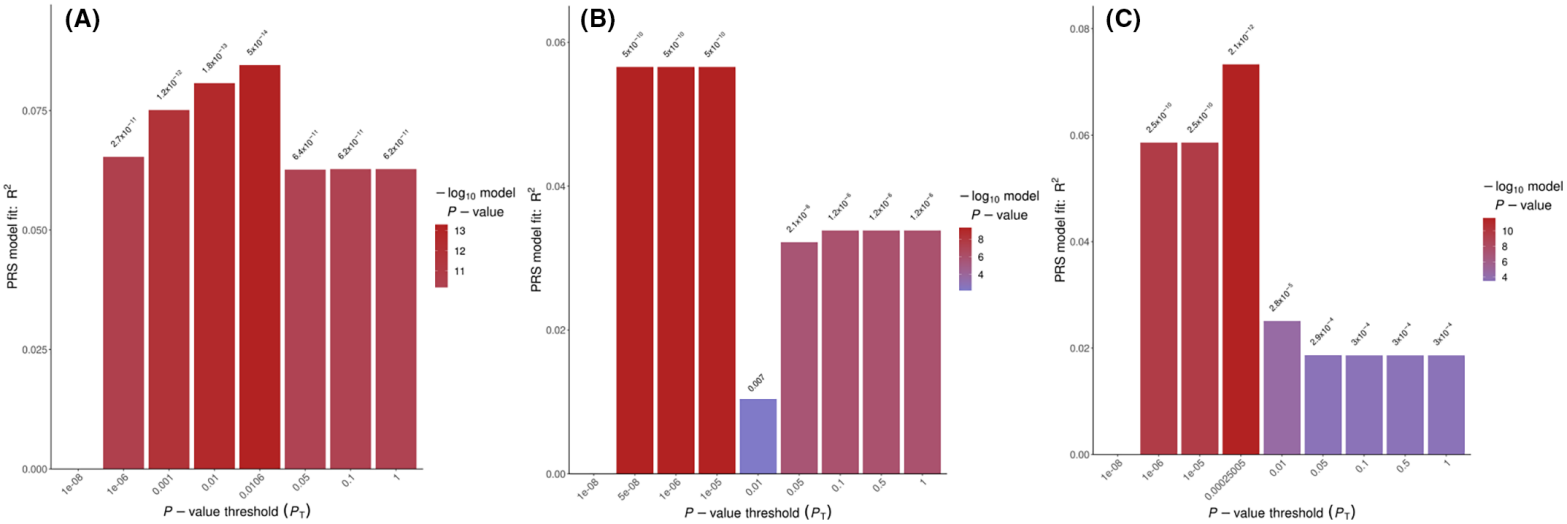
The variance explained by polygenic risk score at different *p*‐value thresholds. (A) East Asian, (B) European, (C) mix ancestral population.

**FIGURE 3:**
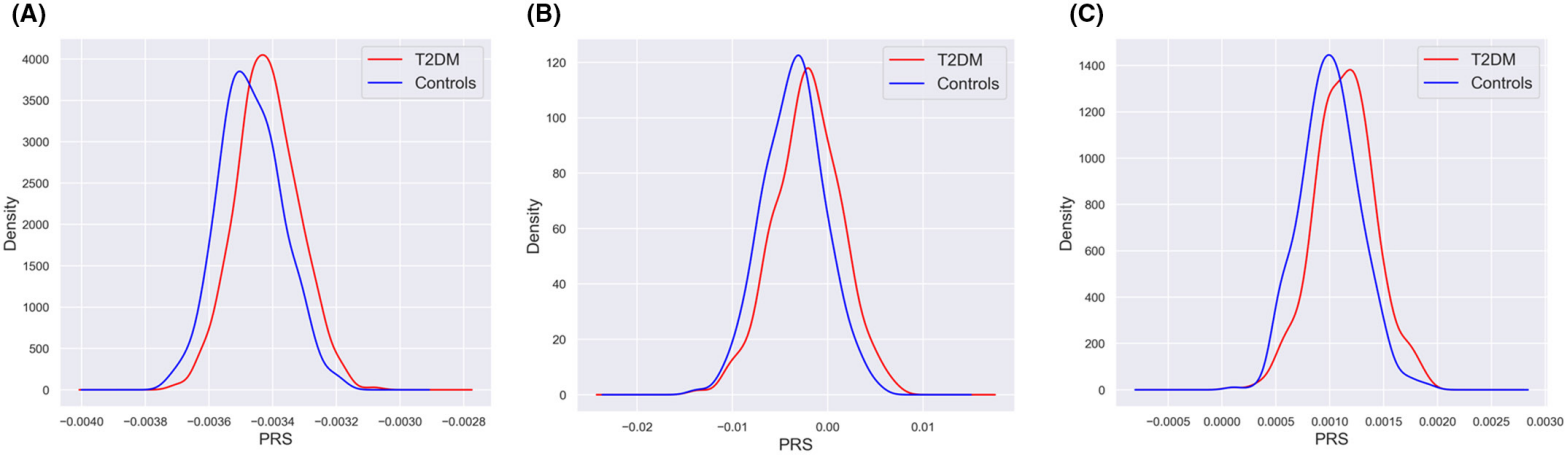
The distribution of PRS among T2DM cases and controls with *p*‐value = 5 × 10^−2^. (A) East Asian, (B) European, (C) mix ancestral population.

The final PRS calculation consisted of 957 individuals (489 T2DM cases and 468 controls), with 7,587,388 SNPs each. The generated PRS was significantly associated with T2DM; however, the predictive capabilities of PRS in the studied population varied with the differences in training datasets. The strongest effect sizes were observed when PRS was calculated from EAS summary statistics, which can explain 8.4% of the variance in T2DM liability (*p*‐value = 5 × 10^−14^). On the other hand, PRS calculated from MIX and EU GWAS summary statistics only accounted for 7.3% and 5.7% of the variability in T2DM risk with *p*‐value = 2.1 × 10^−12^ and 5 × 10^−10^, respectively (Figure 2). The best model for T2DM prediction derived from MIX summary statistics showed AUC = 0.70 (95% confident interval = 0.62–0.77) achieved with a ‘clump’ *p*‐value = 0.05 and 80:20 Training: Testing ratio (Table 2). The distribution of PRS among T2DM cases and controls with *p*‐value = 5 × 10^−2^ is illustrated in Figure 3. The distribution of PRS among T2DM cases and controls with other *p*‐values (5 × 10^−4^, 5 × 10^−6^, 5 × 10^−8^) is shown in Figure S1.

**TABLE 2:** Polygenic risk score calculations based on different training datasets and ratios.

*p*‐value threshold			EAS	EU	MIX
5 × 10^−2^	Number of SNPs	Discovery data	755,536	449,801	799,589
		Target data	723,212	379,281	789,267
	AUC (Training: testing)	20:80 (193:764)	0.66 (0.61–0.69)	0.62 (0.58–0.66)	0.63 (0.59–0.67)
		50:50 (478:479)	0.63 (0.58–0.68)	0.64 (0.59–0.69)	0.64 (0.60–0.69)
		80:20 (762:195)	0.62 (0.54–0.70)	0.63 (0.55–0.71)	**0.70** **(0.62–0.77)**
5 × 10^−4^	Number of SNPs	Discovery data	94,035	19,579	93,689
		Target data	91,222	16,538	91,642
	AUC (Training: testing)	20:80 (193:764)	0.64 (0.60–0.68)	0.62 (0.58–0.66)	0.63 (0.57–0.67)
		50:50 (478:479)	0.63 (0.58–0.68)	0.64 (0.59–0.69)	0.64 (0.60–0.69)
		80:20 (762:195)	0.62 (0.55–0.70)	0.63 (0.55–0.71)	**0.70** **(0.62–0.77)**
5 × 10^−6^	Number of SNPs	Discovery data	30,697	3850	34,909
		Target data	28,912	3261	33,923
	AUC (Training: testing)	20:80 (193:764)	0.63 (0.59–0.67)	0.62 (0.58–0.66)	0.62 (0.58–0.66)
		50:50 (478:479)	0.62 (0.57–0.67)	0.64 (0.59–0.69)	0.62 (0.57–0.67)
		80:20 (762:195)	0.62 (0.54–0.70)	0.63 (0.55–0.71)	0.66 (0.59–0.74)
5 × 10^−8^	Number of SNPs	Discovery data	17,283	1695	17,775
		Target data	14,949	1436	17,672
	AUC (Training: testing)	20:80 (193:764)	0.63 (0.59–0.67)	0.62 (0.58–0.66)	0.61 (0.57–0.65)
		50:50 (478:479)	0.62 (0.57–0.67)	0.64 (0.59–0.69)	0.61 (0.56–0.66)
		80:20 (762:195)	0.61 (0.53–0.69)	0.63 (0.55–0.71)	0.66 (0.58–0.74)

*Note*: Discovery data: data obtained from the GWAS Catalogue of the European Bioinformatics Institute. Target data: data of SNPs which were identical to those in the ‘Discovery data’ and available in our studied data after imputation.

Abbreviations: AUC, area under the curve; EAS, East Asian; EU, European; MIX, mix ancestral.

## 4 DISCUSSION

Genetics play an essential role in the pathophysiology of T2DM; however, defining a genetic model for T2DM is not always feasible due to the heterogeneous nature of the disease.^20^, ^21^ Several attempts have been made to identify the genetic associations between certain loci and T2DM in Kinh Vietnamese,^22^, ^23^ but more information is required for a comprehensive risk prediction model for T2DM.

In this study, the T2DM age at onset was 46, which is relatively young, and this reflected two issues. First, the genetic contribution to T2DM may be greater in this population. Second, the early onset age will create a significant healthcare burden for T2DM in the future.^24^, ^25^ BMI, waist–hip ratio and systolic blood pressure were also significantly greater in the T2DM group compared to the controls. These parameters could be applied to the logistic model to predict T2DM; however, it would be best if the parameters could be obtained before treatment, as several management paradigms of T2DM could affect patient BMI, waist–hip ratio, and systolic blood pressure. There was no BMI criterion used for recruitment in the control and T2DM group. Overweight/obesity is a distinct phenotype and a strong risk factor for T2DM development that can be explained by both genetic and environmental components. Higher BMI observed in the T2DM group, therefore, may result from different genetic characteristics.

With the sample size in this study, we could not detect any ‘truly’ statistically significant SNPs associated with the T2DM phenotype (*p*‐value ≤5 × 10^−8^). It is reasonable to use this cut‐off to avoid a coincident association based on Bonferroni correction, as millions of SNP tests were performed.^26^ However, several authors have suggested that these criteria are too stringent and should be adjusted based on factors such as minor allele frequency or number of permutations.^27^, ^28^, ^29^ When using Benjamini–Hocberg procedure, a less stringent approach, we could identify 37 SNPs statistically associated with T2DM and we did observe a strong signal in chromosome 17. Interestingly, rs11079784 was found to be associated with T2DM both before and after imputation. This SNP is located in the region *KPNB1*‐*DT*, *NPEPPS* gene, which has been reported to be associated with multiple sclerosis.^30^, ^31^ The association between rs11079784 and T2DM, however, remains unclear and needs to be addressed in further studies. The suggestive signal in chromosome 17 once again emphasizes the importance of diverse populations in GWAS of T2DM. The information in this study may not appear of particular significance on its own; however, when incorporated into other populations, it can help in the discovery of association signals shared across different populations and allow fine‐mapping of the causal variants.

PRS calculated from MIX summary statistics performs better than from EAS summary statistics, and both models can predict T2DM more precisely than the model using EU training data. This observation is similar to a study calculating PRS for schizophrenia performed in Kinh Vietnamese in which the MIX ancestral training dataset showed better phenotype prediction.^10^ Using the base GWAS data with larger sample size and more diverse origin seems to increase the PRS's predictive capability. These results suggest the importance of a large trans‐ancestral GWAS database, as it will help create a better predictive model, especially for Southeast Asian populations such as Kinh Vietnamese. The best predictive model for T2DM using only genetic data in this study has AUC = 0.70. The capability of T2DM prediction could be increased by adding clinical data to the model.^32^, ^33^ Ideally, these data should be collected at the time of diagnosis and prior to any treatment. Given the nature of a cross‐sectional study, we could not obtain clinical parameters at the time of diagnosis for all the T2DM cases; therefore, combining clinical parameters in the model to predict T2DM was not appropriate.

Besides future meta‐analysis, another application of these GWAS data is expression quantitative trait loci analysis. Further functional studies on genes' expression affected by T2DM‐associated variants will help better understand the disease biological mechanisms, identify molecular pathways, and even determine potential drug targets.

Although this study has answered our original questions, it has several limitations. First, genetic imputation was performed using the 1000 Genomes database for East Asia due to the absence of specific whole genome data for Kinh Vietnamese; this may not reflect a perfect linkage disequilibrium, as Kinh Vietnamese have distinct genetic characteristics.^34^, ^35^, ^36^, ^37^ Second, the studied sample size is not large enough to demonstrate a ‘truly’ significant SNP for T2DM. Third, the lack of clinical parameters at the time of diagnosis meant that predictive values for T2DM could not be improved.

To the best of our knowledge, this is the first study investigating genome‐wide association and PRS estimation for T2DM in the Kinh Vietnamese population. Although the study has limitations, it could pave the way for larger and more detailed studies to understand better the genetic architecture of T2DM in a Vietnamese population. This understanding will help reduce healthcare disparities as well as enable better prediction and prevention of T2DM in Vietnam.

## AUTHOR CONTRIBUTIONS

**Thao Phuong Mai:** Conceptualization (equal); formal analysis (equal); investigation (equal). **Bac An Luong:** Formal analysis (equal); investigation (equal); methodology (equal); software (equal); validation (equal). **Phat Tung Ma:** Data curation (equal). **Thang Viet Tran:** Data curation (equal). **Tat Thang Dinh Ngo:** Data curation (equal). **Chi Khanh Hoang:** Data curation (equal). **Luong Van Tran:** Data curation (equal). **Bao Hoang Le:** Data curation (equal). **Hoang Anh Vu:** Investigation (equal); validation (equal). **Linh Hoang Gia Le:** Investigation (equal). **Khuong Thai Le:** Investigation (equal). **Steven Truong:** Conceptualization (equal); formal analysis (equal). **Nam Quang Tran:** Conceptualization (equal); data curation (equal); methodology (equal). **Minh Duc Do:** Conceptualization (equal); formal analysis (equal); funding acquisition (equal); methodology (equal); project administration (equal); resources (equal); supervision (equal); validation (equal); writing – original draft (equal); writing – review and editing (equal).

## FUNDING INFORMATION

This research is funded by Vietnam National Foundation for Science & Technology Development (NAFOSTED) under grant number 108.01‐2019.319.

## CONFLICT OF INTEREST STATEMENT

The authors report no conflicts of interest in this work.

## Supporting information


**Figure S1.** The distribution of PRS among T2DM cases and controls with *p*‐value = 5 × 10^−4^, 5 × 10^−6^, 5 × 10^−8^. A: East Asian, B: European, C: mix ancestral population.

Table S1.

## Data Availability

The datasets used and/or analysed during the current study are available from the corresponding author on reasonable request: please contact ducminh@ump.edu.vn.
