# Supplementary figures and images for: Genome‐wide association and polygenic risk score estimation of type 2 diabetes mellitus in Kinh Vietnamese—A pilot study
Source: J Cell Mol Med. 2024 Jul 3;28(13):e18526. doi: 10.1111/jcmm.18526 (PMC11220366; doi:10.1111/jcmm.18526)

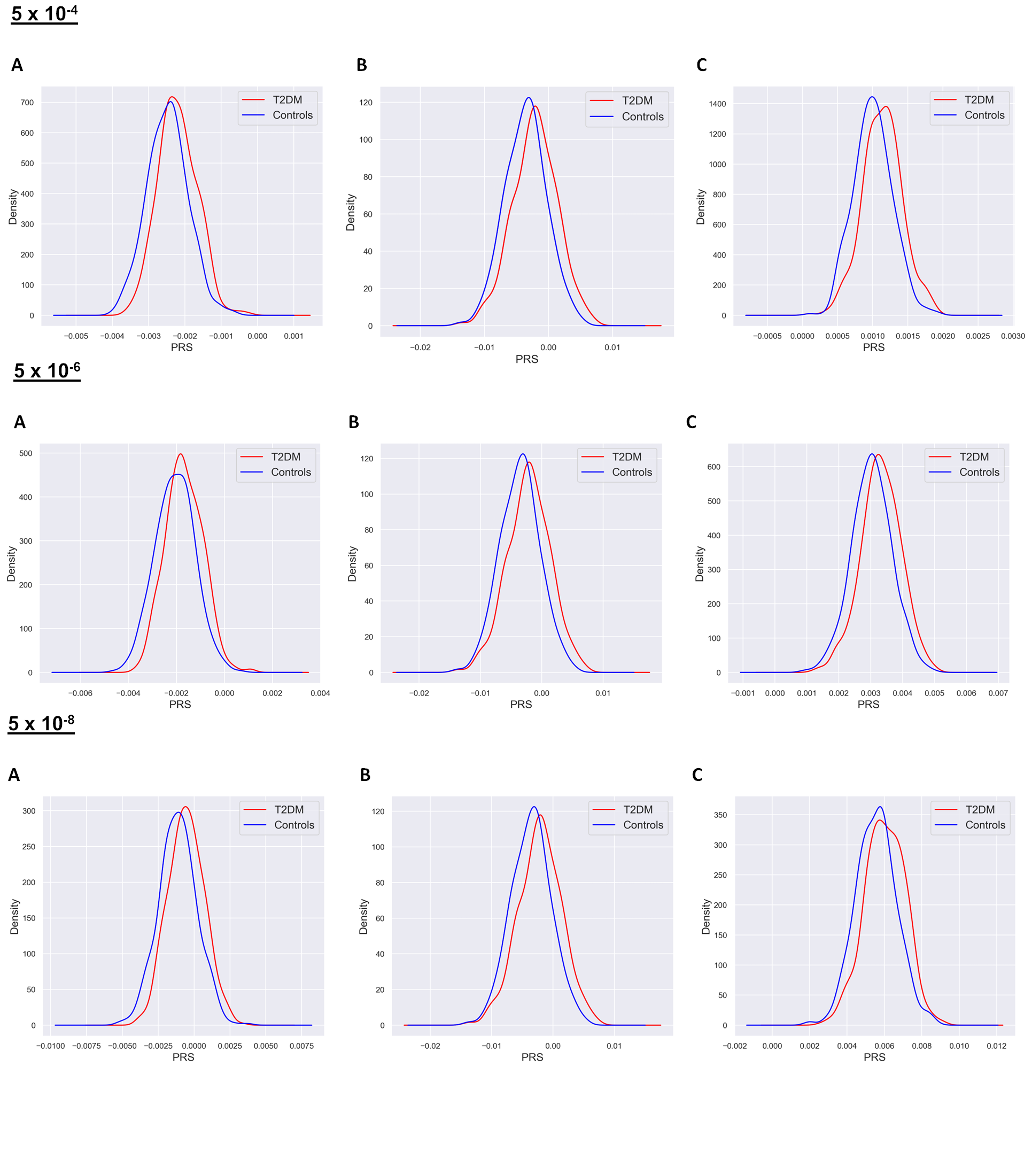

Supplement: Supplementary file 1 — Figure S1. The distribution of PRS among T2DM cases and controls with p‐value = 5 × 10^−4^, 5 × 10^−6^, 5 × 10^−8^. A: East Asian, B: European, C: mix ancestral population. [file JCMM-28-e18526-s002.tif]
