# Supplementary material for: Genome‐wide association and polygenic risk score estimation of type 2 diabetes mellitus in Kinh Vietnamese—A pilot study
Source: J Cell Mol Med. 2024 Jul 3;28(13):e18526. doi: 10.1111/jcmm.18526 (PMC11220366; doi:10.1111/jcmm.18526)
Supplement: Supplementary file 2 — Table S1. [file JCMM-28-e18526-s001.docx]

Supplementary table 1. SNPs associated with T2DM in the studied population by ascending P-value order.

| SNP_ID | CHR | BP | A1 | A2 | Impute score | MAF | OR | P | Rank | Benjamini-Hochberg correction p-value | Statistical significance (B-H procedure) |
| --- | --- | --- | --- | --- | --- | --- | --- | --- | --- | --- | --- |
| rs200908868 | 17 | 45575934 | A | G | 0.951594 | 0.415497 | 1.57298 | 8.27E-07 | 1 | 2.64E-08 | No |
| rs62076549 | 17 | 45579555 | T | G | 0.985882 | 0.430563 | 0.632892 | 8.36E-07 | 2 | 5.27E-08 | No |
| rs7757021 | 6 | 16111361 | C | T | 0.904369 | 0.490663 | 1.54141 | 8.78E-07 | 3 | 7.91E-08 | No |
| rs6432832 | 2 | 1.66E+08 | A | C | 0.982049 | 0.164756 | 1.82047 | 9.77E-07 | 4 | 1.05E-07 | No |
| rs13426374 | 2 | 1.66E+08 | T | C | 0.982189 | 0.164709 | 1.82094 | 9.80E-07 | 5 | 1.32E-07 | No |
| rs72228483 | 2 | 1.66E+08 | CA | C | 0.981566 | 0.167885 | 1.80813 | 1.07E-06 | 6 | 1.58E-07 | No |
| rs12103836 | 17 | 45577469 | G | A | 0.98431 | 0.430004 | 1.56994 | 1.15E-06 | 7 | 1.85E-07 | No |
| rs4794051 | 17 | 45577914 | A | G | 0.984453 | 0.429964 | 1.56937 | 1.17E-06 | 8 | 2.11E-07 | No |
| rs141516856 | 17 | 45576936 | C | G | 0.983779 | 0.430138 | 1.56913 | 1.17E-06 | 9 | 2.37E-07 | No |
| rs2222000 | 2 | 1.66E+08 | G | A | 0.980932 | 0.164056 | 1.81382 | 1.18E-06 | 10 | 2.64E-07 | No |
| rs3865314 | 17 | 45669524 | A | C | 0.980878 | 0.441976 | 0.642287 | 1.23E-06 | 11 | 2.90E-07 | No |
| rs11870057 | 17 | 45588703 | G | A | 0.982893 | 0.431093 | 0.638542 | 1.23E-06 | 12 | 3.16E-07 | No |
| rs35159316 | 17 | 45590257 | C | T | 0.9832 | 0.431041 | 0.638541 | 1.24E-06 | 13 | 3.43E-07 | No |
| rs11869940 | 17 | 45578435 | T | A | 0.98397 | 0.429752 | 0.638104 | 1.25E-06 | 14 | 3.69E-07 | No |
| rs11212752 | 11 | 97665057 | T | C | 0.972283 | 0.386363 | 0.64194 | 1.25E-06 | 15 | 3.95E-07 | No |
| rs7423330 | 2 | 1.66E+08 | G | T | 0.982887 | 0.164809 | 1.8114 | 1.29E-06 | 16 | 4.22E-07 | No |
| rs1138193 | 17 | 45651056 | C | T | 0.992767 | 0.435752 | 0.63984 | 1.32E-06 | 17 | 4.48E-07 | No |
| rs200579989 | 17 | 45655582 | AAGAG | A | 0.993098 | 0.435718 | 0.639851 | 1.32E-06 | 18 | 4.74E-07 | No |
| rs4793836 | 17 | 45670312 | G | A | 0.994312 | 0.435574 | 0.639913 | 1.34E-06 | 19 | 5.01E-07 | No |
| rs12936468 | 17 | 45672200 | G | T | 0.994466 | 0.435555 | 0.639917 | 1.35E-06 | 20 | 5.27E-07 | No |
| rs56969181 | 17 | 45645956 | CA | C | 0.983216 | 0.441195 | 0.643329 | 1.35E-06 | 21 | 5.54E-07 | No |
| rs6505049 | 17 | 45675781 | G | A | 0.994775 | 0.435524 | 0.639934 | 1.35E-06 | 22 | 5.80E-07 | No |
| rs4794007 | 17 | 45590722 | G | A | 0.983892 | 0.43064 | 0.640259 | 1.42E-06 | 23 | 6.06E-07 | No |
| rs8065990 | 17 | 45591166 | G | A | 0.983978 | 0.430623 | 0.640252 | 1.42E-06 | 24 | 6.33E-07 | No |
| rs4794371 | 17 | 45627693 | G | C | 0.99132 | 0.435659 | 0.641092 | 1.42E-06 | 25 | 6.59E-07 | No |
| rs113714334 | 17 | 45601493 | GA | G | 0.989746 | 0.435865 | 0.641487 | 1.44E-06 | 26 | 6.85E-07 | No |
| rs200949448 | 17 | 45601675 | C | CA | 0.979964 | 0.439176 | 0.64327 | 1.45E-06 | 27 | 7.12E-07 | No |
| rs55687600 | 17 | 45630497 | C | T | 0.99182 | 0.435566 | 0.641413 | 1.47E-06 | 28 | 7.38E-07 | No |
| rs74951133 | 17 | 45632955 | T | C | 0.992012 | 0.435543 | 0.641422 | 1.47E-06 | 29 | 7.64E-07 | No |
| rs35806769 | 17 | 45634013 | G | A | 0.992092 | 0.435532 | 0.641425 | 1.47E-06 | 30 | 7.91E-07 | No |
| rs9635762 | 17 | 45648446 | A | C | 0.992961 | 0.435347 | 0.64135 | 1.48E-06 | 31 | 8.17E-07 | No |
| rs8070759 | 17 | 45638760 | C | T | 0.992432 | 0.435487 | 0.641465 | 1.48E-06 | 32 | 8.44E-07 | No |
| rs9891111 | 17 | 45709375 | C | G | 0.99663 | 0.434603 | 1.56032 | 1.48E-06 | 33 | 8.70E-07 | No |
| rs2644349 | 17 | 45641743 | G | A | 0.992663 | 0.435458 | 0.641468 | 1.48E-06 | 34 | 8.96E-07 | No |
| rs8082284 | 17 | 45647067 | T | C | 0.993069 | 0.435411 | 0.641478 | 1.49E-06 | 35 | 9.23E-07 | No |
| rs8072644 | 17 | 45653364 | A | T | 0.993552 | 0.435353 | 0.64151 | 1.50E-06 | 36 | 9.49E-07 | No |
| rs2644364 | 17 | 45653569 | C | T | 0.993565 | 0.435351 | 0.641511 | 1.50E-06 | 37 | 9.75E-07 | No |
| rs79443387 | 17 | 45626961 | C | T | 0.991322 | 0.435496 | 0.641859 | 1.51E-06 | 38 | 1.00E-06 | No |
| rs35794672 | 17 | 45612906 | G | A | 0.990321 | 0.43563 | 0.641997 | 1.51E-06 | 39 | 1.03E-06 | No |
| rs145346039 | 17 | 45638267 | G | C | 0.991771 | 0.434388 | 0.641227 | 1.51E-06 | 40 | 1.05E-06 | No |
| rs10432035 | 17 | 45624913 | G | C | 0.991209 | 0.435506 | 0.641922 | 1.51E-06 | 41 | 1.08E-06 | No |
| rs75283674 | 17 | 45660887 | T | G | 0.994143 | 0.435288 | 0.641525 | 1.51E-06 | 42 | 1.11E-06 | No |
| rs199538746 | 17 | 45665177 | A | T | 0.994499 | 0.435248 | 0.64155 | 1.52E-06 | 43 | 1.13E-06 | No |
| rs4092460 | 17 | 45667550 | G | A | 0.994684 | 0.435226 | 0.641552 | 1.53E-06 | 44 | 1.16E-06 | No |
| rs72829689 | 17 | 45593661 | T | C | 0.987448 | 0.432158 | 0.640575 | 1.53E-06 | 45 | 1.19E-06 | No |
| rs78737171 | 17 | 45668760 | A | T | 0.994789 | 0.435216 | 0.641561 | 1.53E-06 | 46 | 1.21E-06 | No |
| rs9635763 | 17 | 45649694 | C | A | 0.992792 | 0.435513 | 0.641832 | 1.53E-06 | 47 | 1.24E-06 | No |
| rs12937560 | 17 | 45671869 | C | T | 0.995041 | 0.435187 | 0.641555 | 1.53E-06 | 48 | 1.27E-06 | No |
| rs4793842 | 17 | 45671506 | G | A | 0.995011 | 0.435189 | 0.641568 | 1.53E-06 | 49 | 1.29E-06 | No |
| rs35139463 | 17 | 45674533 | TTATAA | T | 0.99527 | 0.435161 | 0.641577 | 1.54E-06 | 50 | 1.32E-06 | No |
| rs8078880 | 17 | 45676526 | G | C | 0.995434 | 0.435146 | 0.64158 | 1.54E-06 | 51 | 1.34E-06 | No |
| rs8075411 | 17 | 45676760 | C | T | 0.995457 | 0.435141 | 0.64159 | 1.54E-06 | 52 | 1.37E-06 | No |
| rs11870585 | 17 | 45618812 | G | A | 0.987958 | 0.436571 | 0.642545 | 1.54E-06 | 53 | 1.40E-06 | No |
| rs4793908 | 17 | 45678408 | G | A | 0.995589 | 0.435126 | 0.641589 | 1.55E-06 | 54 | 1.42E-06 | No |
| rs4793913 | 17 | 45678579 | C | T | 0.995608 | 0.435124 | 0.6416 | 1.55E-06 | 55 | 1.45E-06 | No |
| rs10163469 | 17 | 45687767 | G | A | 0.996399 | 0.435042 | 0.641624 | 1.56E-06 | 56 | 1.48E-06 | No |
| rs12943464 | 17 | 45690351 | A | T | 0.99663 | 0.435019 | 0.641629 | 1.57E-06 | 57 | 1.50E-06 | No |
| rs7214799 | 17 | 45702099 | C | T | 0.994188 | 0.433105 | 0.641397 | 1.57E-06 | 58 | 1.53E-06 | No |
| rs71303319 | 17 | 45694980 | G | GTAAT | 0.997048 | 0.434975 | 0.641642 | 1.58E-06 | 59 | 1.56E-06 | No |
| rs7213086 | 17 | 45752310 | C | G | 0.99555 | 0.435407 | 1.55677 | 1.58E-06 | 60 | 1.58E-06 | Yes |
| rs11871606 | 17 | 45732774 | C | A | 0.997538 | 0.435089 | 0.642213 | 1.58E-06 | 61 | 1.61E-06 | Yes |
| rs3760370 | 17 | 45695832 | C | T | 0.997122 | 0.43497 | 0.641651 | 1.58E-06 | 62 | 1.63E-06 | Yes |
| rs7207542 | 17 | 45697549 | C | G | 0.997307 | 0.434952 | 0.641672 | 1.58E-06 | 63 | 1.66E-06 | Yes |
| rs4793978 | 17 | 45698175 | G | A | 0.997391 | 0.434943 | 0.641661 | 1.58E-06 | 64 | 1.69E-06 | Yes |
| rs11295316 | 17 | 45701027 | AT | A | 0.998307 | 0.434854 | 0.641684 | 1.60E-06 | 65 | 1.71E-06 | Yes |
| rs7219303 | 17 | 45737701 | A | G | 0.997198 | 0.435091 | 0.642588 | 1.62E-06 | 66 | 1.74E-06 | Yes |
| rs11079784 | 17 | 45702280 | T | C | 1 | 0.434692 | 0.641758 | 1.64E-06 | 67 | 1.77E-06 | Yes |
| rs9905922 | 17 | 45740805 | A | G | 0.996988 | 0.435092 | 1.55566 | 1.65E-06 | 68 | 1.79E-06 | Yes |
| rs3874910 | 17 | 45607563 | C | T | 0.972473 | 0.443502 | 0.645409 | 1.66E-06 | 69 | 1.82E-06 | Yes |
| rs7223029 | 17 | 45701731 | T | C | 0.997575 | 0.435187 | 0.642384 | 1.66E-06 | 70 | 1.85E-06 | Yes |
| rs73315878 | 17 | 45744993 | G | A | 0.996731 | 0.435093 | 0.643142 | 1.69E-06 | 71 | 1.87E-06 | Yes |
| rs67114322 | 17 | 45695242 | AT | A | 0.996515 | 0.434808 | 0.642483 | 1.69E-06 | 72 | 1.90E-06 | Yes |
| rs4289035 | 17 | 45707211 | C | T | 0.99938 | 0.43475 | 1.5567 | 1.71E-06 | 73 | 1.92E-06 | Yes |
| rs4399567 | 17 | 45749172 | C | G | 0.996492 | 0.435094 | 0.643462 | 1.73E-06 | 74 | 1.95E-06 | Yes |
| rs34622766 | 17 | 45748897 | TATTA | T | 0.996477 | 0.435102 | 0.643486 | 1.73E-06 | 75 | 1.98E-06 | Yes |
| rs4794058 | 17 | 45597098 | C | T | 0.988249 | 0.431741 | 0.64229 | 1.75E-06 | 76 | 2.00E-06 | Yes |
| rs11656855 | 17 | 45714655 | C | T | 0.999013 | 0.434791 | 0.642827 | 1.76E-06 | 77 | 2.03E-06 | Yes |
| rs10853090 | 17 | 45716124 | C | T | 0.998944 | 0.434798 | 0.642914 | 1.77E-06 | 78 | 2.06E-06 | Yes |
| rs71141922 | 17 | 45715430 | AT | A | 0.997891 | 0.435011 | 0.643253 | 1.79E-06 | 79 | 2.08E-06 | Yes |
| rs55633793 | 17 | 45719816 | G | A | 0.998784 | 0.434818 | 1.5549 | 1.79E-06 | 80 | 2.11E-06 | Yes |
| rs4794001 | 17 | 45720849 | T | C | 0.998709 | 0.434825 | 1.55478 | 1.80E-06 | 81 | 2.14E-06 | Yes |
| rs11444334 | 17 | 45720627 | A | AC | 0.998745 | 0.434821 | 1.55475 | 1.80E-06 | 82 | 2.16E-06 | Yes |
| rs4239162 | 17 | 45755810 | G | A | 0.996016 | 0.435062 | 1.55249 | 1.81E-06 | 83 | 2.19E-06 | Yes |
| rs4239163 | 17 | 45755954 | C | T | 0.996009 | 0.435064 | 1.55246 | 1.81E-06 | 84 | 2.21E-06 | Yes |
| rs140734195 | 17 | 45580138 | C | T | 0.983442 | 0.432263 | 0.643522 | 1.84E-06 | 85 | 2.24E-06 | Yes |
| rs11079779 | 17 | 45640770 | C | T | 0.98873 | 0.437779 | 0.646466 | 1.98E-06 | 86 | 2.27E-06 | Yes |
| rs4794372 | 17 | 45627760 | G | A | 0.991171 | 0.436675 | 0.646701 | 2.15E-06 | 87 | 2.29E-06 | Yes |
| rs72775596 | 10 | 7480937 | T | C | 0.95752 | 0.063574 | 2.58445 | 2.20E-06 | 88 | 2.32E-06 | Yes |
| rs7221548 | 17 | 45582952 | T | C | 0.985544 | 0.431379 | 0.645879 | 2.26E-06 | 89 | 2.35E-06 | Yes |
| rs8074149 | 17 | 45583375 | A | C | 0.985627 | 0.431363 | 0.645874 | 2.26E-06 | 90 | 2.37E-06 | Yes |
| rs12051716 | 17 | 45582395 | G | A | 0.985418 | 0.431393 | 0.645904 | 2.26E-06 | 91 | 2.40E-06 | Yes |
| rs34187971 | 17 | 45649408 | C | CA | 0.982829 | 0.439791 | 0.647655 | 2.30E-06 | 92 | 2.43E-06 | Yes |
| rs58468752 | 17 | 45661289 | T | TA | 0.850265 | 0.476824 | 0.671254 | 2.31E-06 | 93 | 2.45E-06 | Yes |
| rs6432831 | 2 | 1.66E+08 | A | G | 0.980072 | 0.163216 | 1.78437 | 2.40E-06 | 94 | 2.48E-06 | Yes |
| rs11079782 | 17 | 45655887 | A | T | 0.987736 | 0.432527 | 0.648478 | 2.44E-06 | 95 | 2.50E-06 | Yes |
| rs35919601 | 17 | 45657843 | C | T | 0.987887 | 0.432507 | 0.648463 | 2.44E-06 | 96 | 2.53E-06 | Yes |
| rs72823473 | 17 | 45321775 | A | T | 0.964928 | 0.443027 | 0.652075 | 2.67E-06 | 97 | 2.56E-06 | No |
| rs72823474 | 17 | 45321776 | G | C | 0.964931 | 0.443027 | 0.652078 | 2.67E-06 | 98 | 2.58E-06 | No |
| rs73075333 | 7 | 27854994 | A | G | 0.911301 | 0.035714 | 0.305253 | 2.70E-06 | 99 | 2.61E-06 | No |
| rs113155660 | 17 | 45631410 | CT | C | 0.853209 | 0.491298 | 0.672867 | 2.80E-06 | 100 | 2.64E-06 | No |
| rs72775593 | 10 | 7480632 | C | A | 0.967679 | 0.062722 | 2.55528 | 3.31E-06 | 101 | 2.66E-06 | No |
| rs72775594 | 10 | 7480697 | G | C | 0.967462 | 0.067174 | 2.55316 | 3.60E-06 | 102 | 2.69E-06 | No |
| rs6503796 | 17 | 45765249 | T | C | 0.99041 | 0.439385 | 1.52809 | 3.61E-06 | 103 | 2.72E-06 | No |
| rs72775591 | 10 | 7479820 | G | C | 0.964891 | 0.062664 | 2.53869 | 3.71E-06 | 104 | 2.74E-06 | No |
| rs56110544 | 10 | 7498773 | C | T | 0.977647 | 0.060565 | 2.59734 | 3.76E-06 | 105 | 2.77E-06 | No |
| rs59183684 | 17 | 45667822 | CA | C | 0.945076 | 0.431936 | 0.659212 | 4.39E-06 | 106 | 2.79E-06 | No |
| rs35523349 | 17 | 45709286 | C | CA | 0.942896 | 0.405562 | 1.52063 | 4.52E-06 | 107 | 2.82E-06 | No |
